# Multi-Pulse Bound Soliton Fiber Laser Based on MoTe_2_ Saturable Absorber

**DOI:** 10.3390/nano13010177

**Published:** 2022-12-30

**Authors:** Bo Guo, Xinyu Guo, Renlai Zhou, Zhongyao Ren, Qiumei Chen, Ruochen Xu, Wenbin Luo

**Affiliations:** Key Laboratory of In-fiber Integrated Optics, Ministry of Education of China, Harbin Engineering University, Harbin 150001, China

**Keywords:** bound soliton, 2D materials, nonlinear optics, saturable absorber, fiber laser

## Abstract

Bound solitons have become a hot topic in the field of nonlinear optics due to their potential applications in optical communication, information processing and radar systems. However, the trapping of the cascaded bound soliton is still a major challenge up to now. Here, we propose and experimentally demonstrate a multi-pulse bound soliton fiber laser based on MoTe_2_ saturable absorber. In the experiment, MoTe_2_ nanosheets were synthesized by chemical vapor deposition and transferred to the fiber taper by optical deposition. Then, by inserting the MoTe_2_ saturable absorber into a ring cavity laser, the two-pulse, three-pulse and four-pulse bound solitons can be stably generated by properly adjusting the pump strength and polarization state. These cascaded bound solitons are expected to be applied to all-optical communication and bring new ideas to the study of soliton lasers.

## 1. Introduction

Since it was demonstrated, in 1984, soliton lasers and their related phenomena have been a hot research topic in the field of nonlinear optics because of their potential applications in optical communication, information processing and radar systems [1,2]. For decades, this field has developed rapidly and various soliton phenomena have been discovered [3]. So far, besides the conventional solitons, researchers have also obtained other soliton pulses, such as self-similar pulses [4], dissipative solitons [5] and optical rogue waves [6]. However, it should be noted that, for these pulses, only one soliton usually propagates in the laser cavity. Then, a series of questions arise naturally: is it possible for multiple soliton pulses to coexist in a laser cavity? If so, how can these soliton pulses be regulated and controlled? Obviously, the exploration of these problems will help us understand the essence of soliton lasers and bring new possible applications.

In theory, when two or more solitons are very close in time or space, they can interact to form a bound state, which is similar to chemical molecules, called bound solitons or soliton molecules, and can be described by nonlinear Schrodinger equation [7,8,9,10,11,12]. In recent years, bound solitons have attracted great interest from many researchers. For example, Tang et al. experimentally observed bound soliton pulses in fiber lasers based on nonlinear polarization evolution technology [13,14,15,16]. In addition, Seong et al. also obtained bound solitons in a passively mode-locked fiber laser with a figure-eight cavity [17]. Recently, Pang et al. and He et al. demonstrated all-optical bit storage in fiber lasers through optically mechanically bound solitons [18,19], respectively. These efforts have deepened our understanding of bound solitons. It can be found that a pulse-shaping device is usually required to obtain bound solitons in fiber lasers. So far, there are mainly two kinds of pulse-shaping technologies, active and passive. Compared with the active scheme [20], the passive scheme, including nonlinear polarization rotation [21,22,23,24,25,26,27,28,29,30], nonlinear amplifying ring mirror [31,32,33], nonlinear multimode interference [34], stimulated Raman scattering [35] and saturable absorber [36,37,38,39], is considered to be a more promising and efficient way to achieve pulse shaping and obtain bound solitons. It should be noted that most of the bound solitons mentioned above are composed of two pulses. How to obtain more pulse bound states is a scientific problem worth exploring. Theoretically, the accumulation of a large number of soliton pulses is a prerequisite, which can be achieved by increasing the pump intensity or the nonlinear effect in the laser cavity [40,41,42]. This is because of the interaction between the bandwidth constraint of the laser cavity and the energy quantization effect and the soliton splitting only occur when the pump power or nonlinearity is high enough [15]. Thus, it is always the dream of researchers to find optical devices with saturable absorption and high nonlinearity.

In recent years, a new layered material, transition metal dichalcogenides (TMDCs), has received great attention in the fields of physics, chemistry and materials due to its great potential in electronic and optoelectronic applications [43,44,45]. TMDC can be expressed by the formula MX_2_ (M = Mo, W, Ta, V, Nb, Re, Ti, etc; X = S, Se, Te), which is usually used for catalysis and field effect transistors. Interestingly, due to the specific two-dimensional restriction of electronic motion and the lack of interlayer coupling, few-layer TMDCs have direct bandgaps, making their nonlinear optical performance significantly better than that of bulk TMDCs [46,47,48,49,50]. Among them, MoS_2_ and WS_2_ have been widely used in ultrafast lasers in recent years. However, the direct bandgaps of single-layer MoS_2_ and WS_2_ are 1.87 eV and 1.98 eV, respectively, and the corresponding operation wavelengths are ~0.66 μm and ~0.63 μm, respectively. Therefore, it is not suitable for communication applications. Meanwhile, layered MoTe_2_ shows excellent saturable absorption [51,52,53,54,55,56] and high nonlinearity [57,58] (third-order nonlinear optical susceptibility is about 9.96 × 10^−11^ esu) at longer wavelengths, which is suitable for the study of bound solitons.

Here, we demonstrate the generation of cascade bound solitons in an erbium-doped fiber laser with a MoTe_2_-coated fiber taper. The cascaded bound solitons obtained are expected to be used in optical communication, information processing and radar systems.

## 2. Preparation, Characterization and Saturable Absorption of Layered MoTe_2_

In the experiment, the MoTe_2_ nanosheets we used were synthesized by chemical vapor deposition, which is similar to the previous report [53]. Specifically, a thin polymethylmethacrylate (PMMA) film is coated on the upper surface of the MoTe_2_ film, and a MoTe_2_–PMMA composite is obtained. Since the adsorption capacity of MoTe_2_–PMMA is greater than that of MoTe_2_ and the substrate, the MoTe_2_–PMMA composite material can be stripped from the substrate. To this end, we transferred it to acetone to etch off the PMMA film. Thereafter, the MoTe_2_ nanosheets were moved into deionized water and clear MoTe_2_ nanosheets were obtained.

**Figure 1:**
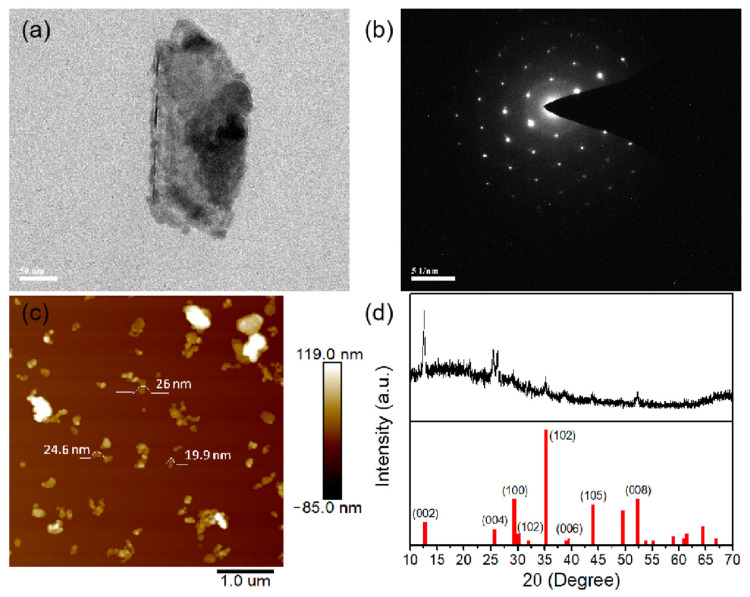
Characterization of few-layer MoTe_2_. (**a**) Transmission electron microscope image; (**b**) electron diffraction patterns; (**c**) atomic force microscopy image and (**d**) X-ray diffraction pattern.

Next, we characterized the MoTe_2_ samples, as shown in Figure 1. Figure 1a is a transmission electron microscope image showing the physical structure of MoTe_2_. It can be seen that most of them are high-quality nanosheets with sharp edges. Figure 1b shows the electron diffraction pattern of MoTe_2_ and a regular array of spots can be observed, indicating that the prepared MoTe_2_ nanosheets have perfect crystal structure. Furthermore, the thickness of the MoTe_2_ nanosheets was characterized by atomic force microscopy, as shown in Figure 1c, ranging from 16 to 26 nm. Since the thickness of a single layer of MoTe_2_ is 0.65 nm, the prepared samples are 25 to 40 layers. The crystal properties of MoTe_2_ nanosheets were characterized by X-ray diffraction device.As shown in Figure 1d, there are four peaks (002), (004), (006) and (008), which indicates that the MoTe_2_ nanosheets are relatively uniform in the direction perpendicular to the sheet plane. These peaks in the X-ray diffraction pattern are designated according to the Joint Committee on Powder Diffraction Standards reference 73-1650. From these observed sharp peaks, it can be determined that MoTe_2_ has a hexagonal crystal structure and its lattice constants are a = 3.52 Å, b = 3.52 Å and b = 13.97 Å, respectively. The relative high strength of the (002) peak indicates that the prepared MoTe_2_ nanosheets have a well-stacked layered structure.

**Figure 2:**
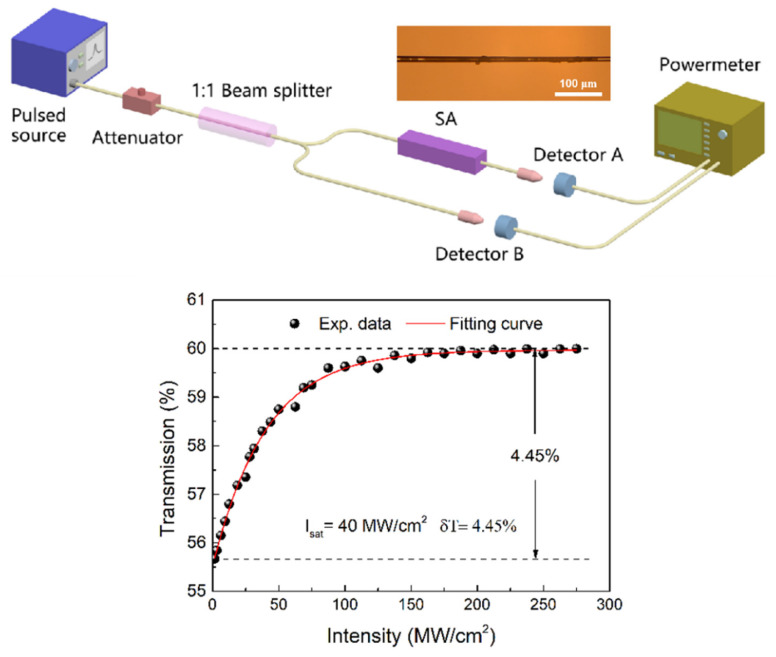
The experimental setup and the corresponding saturable absorption curve of the MoTe_2_-assisted microfiber device (inset: the photograph of the MoTe_2_ device).

Furthermore, we also use power-dependent transmission technology to study the saturated absorption of MoTe_2_-coated microfiber device. The experimental device is shown in Figure 2. Here, the light source used is a femtosecond laser and its parameters are: the center wavelength is 1550 nm, the pulse width is 500 fs, and the repetition frequency is 50 MHz. In the experiment, the optical transmittance under different input light intensities can be obtained by continuously adjusting the output power of the laser, as shown in Figure 2. Similar to graphene, layered MoTe_2_ also has a Dirac cone structure and its saturation absorption is also caused by Pauli-blocking effect. According to the theoretical model [44], the modulation depth, saturation strength and nonsaturable loss of MoTe_2_-assisted tapered fiber devices are about 4.45%, 40 MW/cm^2^ and 40%, respectively. In this experiment, the insertion loss of MoTe_2_-assisted fiber taper is about 3.5 dB. In the future, it is expected to obtain higher quality saturable absorbers by optimizing the fabrication process of fiber taper and the thickness and uniformity of MoTe_2_ nanosheets. As mentioned above, the direct band gap of single-layer MoTe_2_ is about 1.25 eV, and the corresponding operation wavelength is about 993 nm, while the operation wavelength in this work is 1550 nm, showing a slight sub-bandgap absorption phenomenon, which may be caused by defects, two-photon absorption or edge-mode absorption. As shown in Figure 1c, the prepared MoTe_2_ nanosheets exhibit uneven thickness and shape distribution, which may lead to relatively high absorption near 1550 nm.

## 3. Experimental Setup

**Figure 3:**
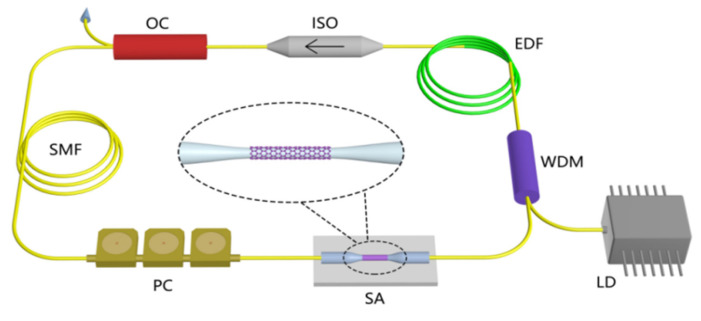
The experimental setup of the proposed fiber laser. LD: laser diode, WDM: wavelength-division multiplexer, EDF: erbium-doped fiber, ISO: polarization-independent isolator, OC: optical coupler, SMF: single-mode fiber, PC: polarization controller, SA: MoTe_2_-assisted fiber taper.

After the preparation of MoTe_2_ nanosheets, we transfer them to the fiber taper by optical deposition method, as shown in Figure 2. The diameter and waist length of the microfiber are about 16 μm and 2 mm. To check the performance of the laser-containing MoTe_2_ device, we insert them into the fiber ring laser cavity, as shown in Figure 3. The laser cavity is composed of 0.5 m highly doped erbium-doped fiber (EDF, Er80-8/125, LIEKKI) and 7.2 m single-mode fiber (SMF), in which the dispersion parameter is 15.7 ps/(km∙nm). As a gain medium, the dispersion parameter is 18 ps/(km∙nm) to improve the beam quality. The total net cavity dispersion is −0.15 ps^2^. Here, the microfiber coated with MoTe_2_ (SA) is inserted into the cavity for pulse shaping. The fiber-pigtail 976 nm laser diode (980-500-B-FA, LD), with the maximum power of 500 mW, is used as the pump light source. In addition, the fused 980/1550 wavelength division multiplexer (WDM) is used to split the light, the 10:90 optical coupler (OC) is used to extract the output of the laser beam, and the polarization-independent isolator (ISO) and polarization controller (PC) are, respectively, used to force the laser to run counterclockwise in one direction and adjust the polarization state in the cavity. The output power, optical spectrum, pulse train and pulse shape of laser pulses are measured by a power meter, a spectral analyzer (ANDO AQ-6317B) with a spectral resolution of 0.01 nm, a photodetector (Thorlabs PDA 12.5 GHz) combined with a 1 GHz hybrid oscilloscope (Tektronix MDO4054-6, 5 GHz/s) and a commercial autocorrelator (APE, PulseCheck), respectively.

## 4. Results and Discussions

Before the soliton experiment, we measured the operation of the laser when there was no MoTe_2_ device and the microfiber was only inserted into the cavity. In the experiment, no matter how the pump power and polarization state of PCs are adjusted, there is only continuous-wave (cw) lasing, thus eliminating the possibility of nonlinear polarization rotation and microfiber mode locking. Then, we insert the MoTe_2_ device into the ring laser cavity, as shown in Figure 3.

**Figure 4:**
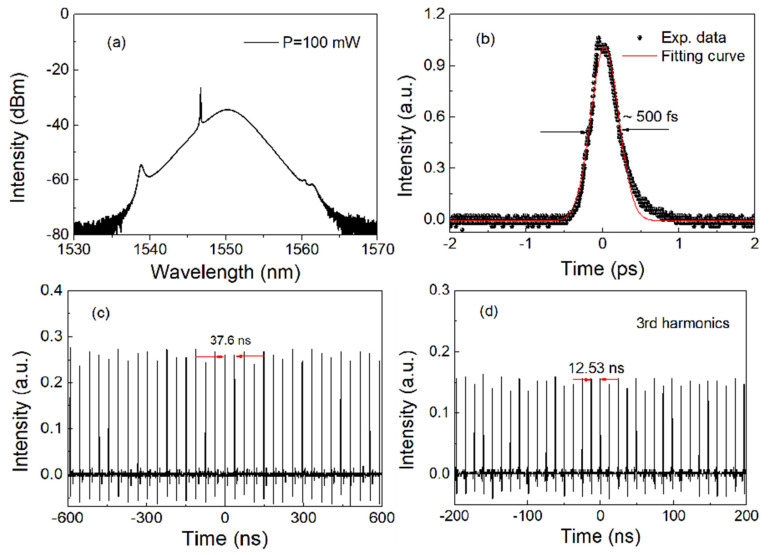
Typical characteristics of conventional soliton pulses: (**a**) optical spectrum; (**b**) autocorrelation trace; (**c**,**d**) oscilloscope’s traces of soliton pulse and its triple form.

Initially, cw operation started at about 50 mW of incident pump power, while self-starting mode-locking operation occurred at about 80 mW. Here, we provide the typical characteristics of soliton pulse in the laser at 100 mW pump power. Figure 4a shows the optical spectrum of the mode-locked pulses. The center wavelength of the pulse is 1550.2 nm and its 3-dB bandwidth is ~5.1 nm. According to the soliton theory [1], there are two pairs of symmetrical Kelly sidebands in the spectrum, indicating that the state is a soliton state. In addition, it can also be observed that there is a peak in the spectrum, which is a continuous wave, which means that the mode-locking is not sufficient [33]. In order to confirm the existence of soliton pulse in the cavity, we need to measure its pulse profile. In the experiment, we use a commercial autocorrelator to measure the autocorrelation trace of soliton pulses on the picosecond time scale, as shown in Figure 4b. If sech^2^ function is used to fit the pulse profile, the pulse width is ~500 fs. By calculation, the time bandwidth product (TBP) of these soliton pulses is about 0.323, close to the theoretical transformation limit (0.315), indicating that the soliton pulses are almost perfect. Figure 4c shows the oscilloscope’s trace of the soliton pulse with a time span of 1.2 μs. It can be seen that the soliton pulse propagates in the cavity with a period of 37.6 ns, which corresponds to the basic repetition frequency of 26.6 MHz. In the experiment, the pulse repetition rate can be increased by increasing the pump power. With the increase in pump power, the oscilloscope displays the pulse sequence within a 0.4 µs range, as shown in Figure 4d. The time interval of the pulse sequence is ~12.53 ns, which corresponds to the repetition frequency of 79.8 MHz, which is three times the basic repetition frequency of the laser, indicating the occurrence of harmonic mode locking. In addition, when the pump power is 100 mW, the output power of the soliton laser is about 5 mW.

**Figure 5:**
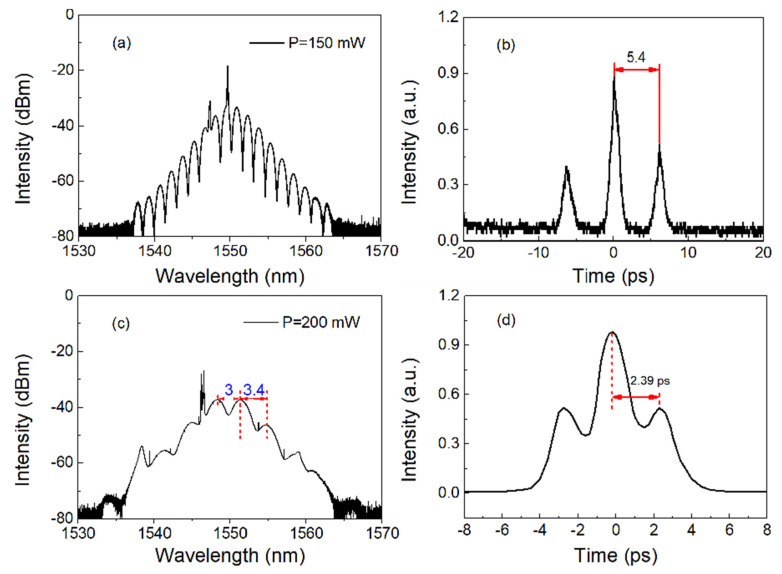
Typical characteristics of two kinds of two-pulse bound solitons: (**a**,**c**) optical spectra; (**b**,**d**) autocorrelation traces.

As we know, bound solitons are multi-pulses with fixed, discrete separation and stable phase difference. To this end, we first make the laser operate in multi-pulse mode. As mentioned earlier, due to the combined effects of the high nonlinearity of MoTe_2_-coated microfiber, the bandwidth constraint of the laser cavity and the energy quantization effect, a single soliton is split into two or more solitons at a higher pump power level, and these solitons are usually randomly located in the cavity. Interestingly, by properly adjusting the pump power and the polarization state in the cavity, they can automatically interact to form a bound soliton state. By properly adjusting the PCs, we can achieve two-pulse bound solitons at 150 mW pump power. Figure 5a shows the spectrum of two-pulse bound solitons with a central wavelength of 1550.2 nm and a modulation period of 1.53 nm. It can be seen that the typical spectrum of the bound soliton is strongly modulated, indicating that the interval between the two-pulse bound solitons is very small. In theory, the soliton separation (Δτ) can be calculated by the equation [59]:

(1) $$\Delta \tau =\lambda_{c}^{2}/(c\cdot \delta \lambda )$$

where c, λ_c_, and δλ are the light speed, center wavelength, and spectral modulation period. In this experiment, these parameters are c = 3 × 10^8^ m/s, λ_c_ = 1550.2 nm, and δλ = 1.53 nm, respectively. Thus, the soliton separation Δτ is about 5.23 ps.

The corresponding autocorrelation trace (AC) shown in Figure 5b shows that it is a two-pulse bound soliton. It can be seen that the amplitude of the secondary peak in the AC is almost half of the amplitude of the main peak. Through calculation, the soliton separation from the autocorrelation trace is 5.4 ps, which is slightly larger than the theoretical value calculated from the above equation. It is worth noting that the separation here is about six times the pulse width of the soliton, which means that there is a strong interaction between the solitons. In the experiment, by increasing the pump power to 200 mW and slightly adjusting the polarization state of PCs, we also observed another two-pulse bound soliton. Figure 5c shows the typical spectrum of two-pulse bound solitons with a center wavelength of 1550.2 nm and a modulation period of 3.4 nm. Similarly, cw and soliton sidebands can be observed. Furthermore, the corresponding AC shown in Figure 5d shows that it is a two-pulse bound soliton. In the experiment, the pulse separation of the soliton is measured to be 2.39 ps, which corresponds to the period of spectral modulation.

**Figure 6:**
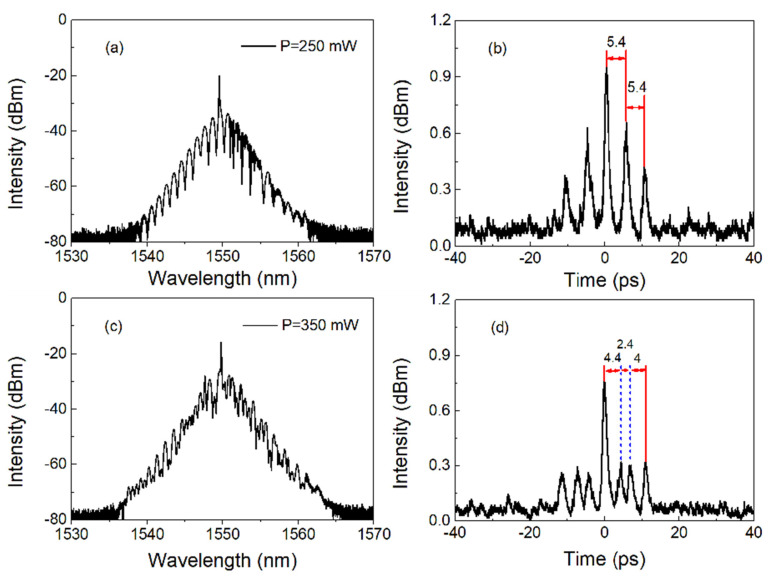
Typical characteristics of three-pulse and four-pulse bound solitons: (**a**,**c**) optical spectra; (**b**,**d**) autocorrelation traces.

In addition, if the pump power is increased from 250 mW to 350 mW and the PC is properly adjusted, three-pulse and four-pulse bound solitons can be obtained, as shown in Figure 6. Figure 6a shows the optical spectrum of a three-pulse bound soliton with a central wavelength of 1550.2 nm and a modulation period of 1.53 nm. It can be seen that the spectrum is strongly modulated. The corresponding AC shown in Figure 6b shows that it is a three-pulse bound soliton. In the experiment, the pulse-to-pulse interval of the soliton is measured to be 5.4 ps, which corresponds to the period of spectral modulation. Figure 6c shows the optical spectrum of a four-pulse bound soliton with 1550.2 nm central wavelength and disorder modulation period. Similarly, it can be seen that the spectrum is strongly modulated. The corresponding AC shown in Figure 6d shows that it is a four-pulse bound soliton. In the experiment, we measured the pulse separation of solitons as 4.4 ps, 2.4 ps and 4 ps, respectively. In this experiment, we notice a hybrid-pulse phenomenon, which is different from the common bound soliton. This may be related to the high nonlinearity of the tapered fiber device, i.e., higher-order effect, which affects the state and interaction of the bound solitons in the laser cavity [40,41]. Further investigation on its formation mechanism is still required in future work.

In the above experiments, we obtained the cascade bound solitons with up to four pulses. This shows that the MoTe_2_-assisted fiber taper is a very good SA with pulse-shaping function, which is suitable for the study of bound solitons. However, as we know, a variety of lasers based on carbon nanotubes [36,37], graphene [38,39,40], topological insulators [41] and black phosphorus [44] can generate bound solitons, which suggests that the appearance of bound solitons is not related to specific materials but a common nonlinear optical phenomenon widely existing in lasers. In this experiment, more bound solitons are obtained due to the fabrication of high-quality fiber taper, which not only provide good mode locking, but also produce high nonlinearity. In particular, the latter results in the splitting of conventional solitons, and then the cascade bound solitons are realized. In theory, the combination of layered materials and fiber taper will greatly enhance the effective third-order nonlinearity in the cavity [60]. Thus, it can be predicted that bound solitons with five or more pulse would be obtained by further optimizing the MoTe_2_-deposited fiber taper and laser parameters, such as polarization state, pump strength and cavity dispersion [61,62,63]. In addition, the cw in the laser may be related to the insufficient modulation depth of the SA. In the future work, we will optimize the preparation of mode-locker to improve the modulation depth and mode locking efficiency.

## 5. Conclusions

In conclusion, we have demonstrated a cascade bound soliton fiber laser based on all-fiber saturable absorber. In the experiment, we obtain two-pulse, three-pulse and four-pulse bound solitons by properly adjusting the pump power and polarization state. For two-pulse bound solitons, the soliton separation is 5.4 ps and 2.39 ps, corresponding to loose and tight binding, respectively. For three-pulse bound solitons, the soliton separation is 5.4 ps. For four-pulse bound solitons, the soliton separation is 4.4 ps, 2.4 ps and 4 ps, respectively, which is the largest number of bound solitons so far generated from passively mode-locked fiber lasers with layered materials. The obtained cascaded bound solitons will be expected to be used in optical telecommunications, information processing and radar systems.

## Data Availability

The data are contained within the article.
